# Commentary: Case report: Mesothelioma and BAP1 tumor predisposition syndrome: implications for public health

**DOI:** 10.3389/fonc.2023.1279786

**Published:** 2023-10-16

**Authors:** Carolina Sassorossi, Marco Chiappetta, Maria Teresa Congedo, Sara Flamini, Annalisa Campanella, Jessica Evangelista, Rodolfo Iuliano, Luigi Boccuto, Filippo Lococo

**Affiliations:** ^1^ Unità Operativa di Chirurgia Toracica, Fondazione Policlinico Agostino Gemelli, Istituto di ricovero e cura a carattere scientifico (IRCCS), Roma, Italy; ^2^ Department of Thoracic Surgery, Istituto di Ricovero e Cura a Carattere Scientifico (IRCCS), Rome, Italy; ^3^ Unità di Genetica Medica, Università Magna Graecia di Catanzaro, Catanzaro, Italy; ^4^ Healthcare Genetics and Genomics, School of Nursing, Clemson University, Clemson, SC, United States

**Keywords:** mesothelioma, surgery, asbestosis, thoracic surgery, BAP1 cancer predisposition syndrome

## Introduction

Pleural mesothelioma is a rare but subtle neoplasm, with a poor prognosis due to a lack of effective and specific treatment. In recent years, the application of next-generation sequencing technology has enabled the discovery of pathogenic gene variants inducing or promoting the development of the disease, alone or in association with environmental exposure to asbestos. In particular, germline variants of *BAP1* may lead to the development of many tumor lesions, mainly uveal melanoma and pleural mesothelioma. In this context, it seems to be clear the role of genetic analysis in families where a potentially disease-causing *BAP1* variant has been suspected. In this context, we read with great interest the article of Vimercati and colleagues (1), who described a case of pleural mesothelioma (PM) in an 83-year-old man, with a story of environmental and working story of asbestos exposure, suspect for BAP1 tumor predisposition syndrome (BAP1-TPDS). Familial history was investigated and specific genetic tests were performed. Based on their results and conclusions and in light of the findings recently reported in a comprehensive review (2), we would like to discuss certain points of their article with a particular focus on the interpretation of genetic findings and the consequent follow-up protocols.

## Discussion and conclusion

Vimercati and colleagues (1) suspected a BAP1 Tumor Predisposition Syndrome (BAP1-TPDS) based on the clinical findings and the patient’s family history. A genetic analysis was conducted on the patient and his son, affected with colon adenocarcinoma.

We would like to focus on the reasons why, in our opinion, the case fails to satisfy the criteria for the diagnosis of typical BAP1-TPDS, and the characterization of the clinical and genetic profiles in case reports of suspected BAP1-TPDS should be at least integrated with more genetic information about the patients and their relatives.

Concerning patient and family history, the patient had a personal anamnesis of uveal melanoma and clear cell renal carcinoma. He also had personal anamnesis of environmental and professional asbestos exposure. He was diagnosed with pleural effusion when he was 83 years old, followed by the diagnosis of mesothelioma after surgery. Firstly, we observe that the age of presentation of PM is not consistent with a BAP1-related PM, since the mean age of mesothelioma onset is around 55 years in these cases (2). It has to be noticed that in the paper of Carbone and colleagues, it is reported a case of BAP1-associated PM in a 84 year-old man, even though, as the author report, this information come from observation not published (3) The patient’s son had a diagnosis of colorectal adenocarcinoma at 32 years and the familial anamnesis was positive for liver carcinoma, laryngeal carcinoma, and lung cancer. We would like to point out that, as reported in our review and by Rai and colleagues (2–4), BAP1-TPDS increases the risk of five major malignancies: uveal melanoma, pleural mesothelioma, atypical intradermal benign tumor with *BAP1*-mutated melanocytes, cutaneous melanoma, and renal cell carcinoma. The syndrome also increases the risk for hepatocellular carcinoma, cholangiocarcinoma, and meningioma. In addition, 90% of patients have a family history of at least two of these tumors. Therefore, the patient was affected with three of these tumors, and even though the mesothelioma had a very late onset, none of his relatives had a diagnosis of the described neoplasm. Furthermore, the long-lasting exposure, both environmental and professional, to asbestos is likely to be the cause of the onset of the mesothelioma. Patient also spent one year in Casale Monferrato well known in Italy for the presence of Eternit (a company producing asbestos cement).

Regarding the histology, on the pathological specimens, a definitive diagnosis of biphasic mesothelioma was made, while mesotheliomas related to BAP1-TPDS are almost exclusively epithelioid type (1), which are less aggressive and are associated with a slightly better prognosis. On a minor note, we would like to point out that the descriptions of Figures 3 and 4 (1) are inverted in the text and may generate confusion.

Concerning the genetic analysis, FISH-analysis was performed on the specimens, after pleurectomy, and revealed the deletion in the 9p21 locus of the *CKN2A* gene in 20% of neoplastic cells. A blood sample of the patient was analyzed too. The test did not detect duplications or deletions of one or more exons or the entire gene and a low percentage of mosaicisms and epimutations. The analysis revealed a heterozygous missense variant in exon7 of *BAP1*, c.535C>T, leading to the substitution of the arginine residue in position 179 with tryptophan. The change is classified as a variant of uncertain significance (VUS) accordingly to the ClinVar database and the ACMG guidelines. It is noteworthy that the variant is not reported in any database, indicating it has never been detected in controls as well as patients: the absence of Mutation/Minor Allele Frequency (MAF) alone is not a strong indicator of pathogenicity, as it constitutes only a moderate parameter for pathogenicity (PM2). However, it suggests that further studies are needed to further characterize the variant. In our opinion, there is no sufficient evidence to support the causative role of this pathogenetic germline mutation. In the genetical tree figure of Vimercati et al., two sons are presented as carriers of *BAP1* mutations. The second mutated son, however, is not taken in consideration in the article (indeed as Vimercati and colleagues state at page 3, no other VUS or germline mutations were found in any member of the family group analysed) and we know nothing about his age. In our opinion, the definition of “familial cluster of *BAP1*-mutation” is wrong as in genetic terminology “cluster” means specifically a group of two or more genes encoding for similar polypeptides. We also noticed some imprecise definition, VOUS instead of VUS, *CDK2A* instead of *CKN2A*. We would encourage the implementation of the use of italics characters for gene names, as suggested by the Sequence Variants Nomenclature (http://varnomen.hgvs.org/).

As discussed in our review (2), BAP1-TPDS is characterized by an autosomal dominant pattern of inheritance with incomplete penetrance. Genetic testing should be considered for all first-grade relatives. The surveillance protocol for carriers of germline *BAP1* pathogenic variants should be extended to the other types of cancers reported in BAP1-TPDS.

Unfortunately, the involvement of germline variants in genes other than *BAP1* is not as deeply characterized, and the available information is insufficient to develop and implement guidelines for tailored genetic screening and/or clinical surveillance.

This case is of great interest in the study of genetic implication in mesothelioma development and the difficult definition of involved variants reflects the gaps in knowledge in this topic. It is a rare disease and even rarer are the cases associated with germline mutations, so not all the *BAP1* pathogenic variants are still known. In case like the one describe further functional validation, may suggest additional types of cancer associated with *BAP1* variants, expanding eventually the phenotype spectrum of BAP1-TPDS, and/or novel pathogenic mechanisms: for these reasons, we would suggest deeper clinical and genetic characterizations in studies focused on cases with suspected BAP1-TPDS. Further studies should be encouraged to acquire more data about germline or somatic mutation, and to define, where possible, a targeted therapy. Furthermore, it is necessary to validate guidelines for genetic screening and surveillance for clusters in which BAP1-TPDS is defined.

## Author contributions

CS: Conceptualization, Data curation, Writing – original draft, Writing – review & editing. MC: Conceptualization, Data curation, Investigation, Writing – review & editing. TC: Conceptualization, Data curation, Methodology, Supervision, Writing – original draft. SF: Conceptualization, Investigation, Writing – review & editing. AC: Conceptualization, Writing – original draft. JE: Writing – review & editing. RI: Conceptualization, Data curation, Investigation, Resources, Writing – original draft. LB: Conceptualization, Data curation, Writing – review & editing. FL: Conceptualization, Data curation, Methodology, Project administration, Writing – review & editing.
